# Difluoromethylornithine modulates mutant *K-RAS* signaling pathways in pancreatic cancer cells

**DOI:** 10.1007/s00726-026-03559-5

**Published:** 2026-09-16

**Authors:** Hwudaurw Chen, David E. Stringer, Haiyan Cui, Eugene W. Gerner, Natalia A. Ignatenko

**Affiliations:** 1 University of Arizona Cancer Center, Tucson, AZ USA; 2 Department of Cellular & Molecular Medicine, University of Arizona, Tucson, AZ USA

**Keywords:** Pancreatic adenocarcinoma, Polyamines, Difluoromethylornithine, Hypusinated eIF5A, RAS-like small GTPase A, Pancreatic orthotopic mouse model

## Abstract

Oncogenic mutations in the K-RAS small GTPase occur in more than 90% of human PDACs and cause dysregulation of multiple metabolic pathways, including polyamine metabolism. The goal of this study was to determine whether blocking polyamine synthesis with the irreversible inhibitor α-difluoromethylornithine (DFMO) provides a valid approach to targeting metabolic dependency in mutant K-RAS pancreatic tumors, compared with direct suppression of the oncogenic K-RAS protein. The anti-tumor activity of DFMO was evaluated *in vitro* and *in vivo* in the MIA PaCa-2 pancreatic cell line (*K-RAS*^*G12C*^) and its isogenic clonally selected line M-27, which does not express a functional GTP-bound RAS protein. MIA PaCa-2 and M-27 cells had comparable intracellular polyamine levels, and DFMO treatment effectively suppressed total polyamine levels in both cell lines (*p* < 0.01). DFMO reduced the protein levels of phosphorylated extracellular signal-regulated kinases 1 and 2 (ERK1/2) and RAS-like small GTPase A (RalA) but induced the phosphoinositide 3-phosphate kinase (PI3K) signaling pathway. Additionally, the intracellular levels of K-RAS and hypusinated eukaryotic initiation factor 5 A (eIF5A^Hyp^) proteins were suppressed in DFMO-treated MIA PaCa-2 cells. DFMO therapy regimen (500 mg/kg/day by oral gavage) significantly improved survival rates of SCID mice with orthotopically implanted MIA PaCa-2 cell line (MIA PaCa-2 control vs. MIA PaCa-2 DFMO, *p* = 0.0184), with median survival time comparable to animals implanted with M-27 cell line (MIA PaCa-2 control vs. M-27 control, *p* = 0.0204). DFMO treatment did not significantly improve survival of mice bearing M-27 tumors (M-27 control vs. M-27 DFMO, *p* = 0.6055). These data indicate that DFMO treatment suppresses pancreatic tumorigenesis driven by the mutant *K-RAS* oncogene similarly to the targeted elimination of the active GTP-bound RAS protein.

**Supplementary Information:**

The online version contains supplementary material available at https://doi.org/10.1007/s00726-026-03559-5.

## Introduction

Pancreatic adenocarcinoma (PDAC) is characterized by its aggressive local invasion of adjacent structures, perineural invasion, desmoplastic stromal reaction, and early lymph and liver metastases (Khan et al. 2021). Mutations in the *K-RAS* oncogene is the most common genetic feature of PDAC, which is required for the progression and maintenance of pancreatic cancer (Khan et al. 2021; Longnecker and Terhune 1998; Lu and Zeng 2017). K-RAS mutations are present in over 90% of PDAC, among which the more frequently identified mutations are *K-RAS*^*G12D*^ (40% of all cases) and *K-RAS*^*G12V*^ (30% of all cases), while the *K-RAS*^*G12C*^ mutations occur in 1–2% of all cases (Luo 2021). The amino acid alterations in the mutated RAS protein disrupt GTP hydrolysis, which is stimulated by the intrinsic GTPase-activating protein (GAP), and lead to accumulation of a high level of the oncogenic, GTP-bound RAS protein in cancer cells (Pylayeva-Gupta et al. 2011). Oncogenic K-RAS transduces different proliferation-stimulating signals and influences cellular processes via a diverse spectrum of downstream effectors, including extracellular signal-regulated kinases (ERK1/2), RAC family small GTPases (RAC GTPase), protein kinase B (AKT), phosphoinositide 3-phosphate lipid kinases (PI3K), and Ras-like (Ral) Guanine Exchange factors (GEFs) (Huang et al. 2021).

Targeting the mutated K-RAS protein and its signaling pathways is an important goal for improving the survival of PDAC patients. A small number of targeted therapies with small-molecule inhibitors of mutant *K-RAS*^*G12C*^ protein have had mixed success, because they were limited to metastatic PDAC patients with rare molecular subtypes and failed to significantly improve patients’ survival (Hu and O’Reilly 2024). Specifically, the use of oral K-RAS mutant-selective inhibitors Sotoprasib and Adagrasib, which target the inactive GDP-bound *K-RAS*^*G12C*^ protein with high specificity (Ostrem et al. 2013; Bar-Sagi et al. 2020), was associated with the development of drug resistance due to accumulation of secondary oncogenic mutations in the *K-RAS* and other genes (Awad et al. 2021; Koga et al. 2021). The significant improvement in targeting *K-RAS* mutations in PDAC has been achieved with the development of a novel multiselective, tri-complex inhibitor of the active GTP-bound RAS protein (RAS (ON) inhibitor), daraxonrasib (Holderfield et al. 2024). A recently conducted Phase 3 clinical trial with daraxonrasib in PDAC patients who previously received conventional chemotherapy for metastatic disease resulted in significantly longer overall survival and progression-free survival than chemotherapy (O’Reilly et al. 2026). But the heterogeneity and adaptability of pancreatic tumors may present a challenge even for RAS (ON) inhibitors, as resistance to novel treatments may develop due to prolonged suppression of both mutant and wild-type RAS activity. Therefore, it is important to continue developing and validating anti-tumor agents targeting cancer cells’ addiction to the oncogenic RAS.

The polyamines spermidine, spermine, and diamine putrescine are polycations that are necessary for normal cellular growth as well as tumorigenesis (Igarashi and Kashiwagi 2000; Gerner and Meyskens 2004). The pancreas contains high amounts of polyamines, especially spermidine (Hyvonen et al. 2007; Hougaard and Larsson 1986), and their biosynthesis is essential for the growth and differentiation of the pancreas (Mastracci et al. 2015). It has been well established that RAS activation in different malignancies, such as colon, skin carcinogenesis, and prostate, leads to oncogenic alterations in polyamine metabolism (Gerner and Meyskens 2004). Furthermore, clinical studies evaluating DFMO, an inhibitor of ornithine decarboxylase (ODC), a rate-limiting polyamine biosynthetic enzyme, in patients with risk factors for cancers of the colon (Meyskens et al. 2008), prostate (Simoneau et al. 2008), and skin (Bailey et al. 2010; Kreul et al. 2012) suggested that DFMO might be a successful strategy to prevent these cancers. As a polyamine-depleting drug, DFMO is approved for the treatment of trypanosomiasis (Bacchi et al. 1980; Milord et al. 1992), hirsutism (Wolf et al. 2007), and neuroblastoma (Oesterheld et al. 2024). Elevated activity of ODC and high polyamine content have been reported in pancreatic cancer cell lines and in the tumor samples of pancreatic cancer patients (Black and Chang 1982; Subhi et al. 2004). Inhibition of the high ODC activity was found to be beneficial in pancreatic tumors with loss of methylthioadenosine phosphorylase (*MTAP*) gene, which encodes for a key catabolic enzyme that catalyzes the cleavage of methylthioadenosine, a byproduct of polyamine biosynthesis (Subhi et al. 2004). DFMO treatment suppressed ODC signaling, cell proliferation, and the pancreatic intraepithelial neoplasia (PanIn) progression in the genetically engineered mouse (GEM) model of pancreatic cancer with the pancreas-specific expression of the mutant K-RAS oncogene (*p48*^*Cre/+;*^*LSL-K-ras*^*G12D/+*^ mouse model) (Mohammed et al. 2014).

Previous studies in neuroblastoma and breast cancer cells identified several mechanisms underlying the anti-tumor activity of DFMO, such as accumulation of the cyclin-dependent kinase inhibitor p27Kip1, and downregulation of the proteins of signal transduction pathways, e.g. c-MYC, phosphorylation of protein kinase B (AKT), glycogen synthase kinase 3β (GSK3-β), and extracellular signal-regulated kinases 1 and 2 (ERK1/2) (Koomoa et al. 2013; Manni et al. 2004). In colon cancer cells, DFMO treatment induced expression of a tumor-suppressive microRNA (miRNA) miRNA *let*7i, which regulates the cell growth-associated transcription factors HMGA2 and LIN28, thus demonstrating that DFMO can suppress tumorigenesis through modulation of miRNA expression (Paz et al. 2014).

In this study, we assessed the anti-tumor efficacy of DFMO in the MIA PaCa-2 human pancreatic cancer cell line. The MIA PaCa-2 cell line is commonly employed for *in vitro* and *in vivo* studies of PDAC tumorigenesis because the morphological and genetic characteristics of this cell line are well characterized. MIA PaCa-2 carries the homozygous missense mutation in codon 12 of exon 2 in the *K-RAS* oncogene, which replaces the amino acid glycine with cysteine (GGT-> TGT) (*K-RAS*^*G12C*^) within the phosphate-binding loop of the K-RAS protein (Moore et al. 2001; Gradiz et al. 2016). Due to this genetic alteration, the MIA PaCa-2 cell line was a particularly suitable model for *in vitro* evaluation of mutant *K-RAS*^*G12C*^*-*selective RAS (OFF) inhibitors (Riedl et al. 2025).

We also utilized a previously characterized MIA PaCa-2 isogenic cell line M-27, which lacks the active GTP-bound form of RAS, thus modeling the molecular effects of RAS (ON) inhibition (Padavano et al. 2015). Comparison of the DFMO-induced changes in K-RAS signaling pathways in MIA PaCa-2 and M-27 cell lines revealed that DFMO effectively targeted the downstream components of RAS-ERK1/2 and RalA signaling pathways in MIA PaCa-2 cells. This resulted in suppression of MIA PaCa-2 cell migration, invasion *in vitro* and increased survival rates of tumor-bearing animals treated with DFMO, an outcome similar to that observed in the M-27 cell line.

## Materials and methods

### Materials

All cell culture reagents used in this study were purchased from Life Technologies, Inc. (Carlsbad, CA). DL-α-Difluoromethylornithine monohydrochloride monohydrate (DFMO, product number D193) was purchased from MilliporeSigma (Burlington, MA, USA). LY294002 (cat. No. S1105) was purchased from Selleck Chemicals LLC (Houston, TX, USA).

### Cell lines

The MIA PaCa-2 human pancreatic cancer cell line (homozygous *K-RAS*^*G12C*^ mutation, homozygous missense P53 mutant, homozygous CDKN2A/p16^INK4A^ deletion) was purchased from American Type Culture Collection (ATCC, Manassas, VA) and was maintained in RPMI-1640 supplemented with 10% fetal bovine serum and 1% penicillin/streptomycin, as well as 0.1% glucose. The Mia PaCa-2 isogenic cell line M-27 expressing a non-functional K-RAS protein due to disruption of *K-RAS*^*G12C*^ mutation via homologous recombination has been previously characterized (Padavano et al. 2015). M-27 cell line was maintained in the same media as MIA PaCa-2 cell line with supplementation of 350 µg/ml G418 neomycin. The cell lines were cultured at 37 °C in a humidified atmosphere of 5% CO2/95% air.

### Growth curve

The MIA PaCa2 cells were plated in six-well plates at a concentration of 0.3 × 10^6^ cells/well in triplicate for each time point (24, 48, 72, and 96 h after subculture) and treated with DFMO at a concentration of 2.5 mM or vehicle (H_2_O) control. The drug was added to the plates at the time of subculture. The cells were harvested and counted using an automated Trypan blue dye exclusion method on a Beckman Coulter Vi-Cell™ Cell Viability analyzer (Beckman Coulter Inc, Miami, FL).

### Polyamine content

Cell extracts were prepared in 0.1 N HCl at a concentration of 4 × 10^7^ cells per 900 µl. Samples were sonicated, adjusted to 0.2 N HCLO_4_, and incubated on ice for at least 2 h. After centrifugation at 13 000 x g for 10 min at 4 °C, the supernatant was assayed for polyamine content by reverse-phase high-performance liquid chromatography with 1,7-diaminoheptane as an internal standard (Seiler 1980). Amines detectable by this method included putrescine, cadaverine, histamine, spermidine, spermine, and monoacetyl derivatives of putrescine, spermidine and spermine, with a limit of detection of 5 pmol. Protein content in each sample was determined by the BCA protein assay kit (Pierce, Rockford, IL). Data are expressed as nmol polyamine per mg protein.

### Membrane isolation

Two 100 mm plates per MIA PaCa-2 control and DFMO-treated at the time of subculture at the concentration of 2.5mM or 5 mM were plated with 1 × 10^6^ cells per plate. The cells were collected at 48 and 96 h after subculture. The membrane extractions were done using MEM_PER™ Plus membrane Protein extraction Kit (ThermoScientific, Cat.# 89842) per the manufacturer’s protocol.

### Western analysis

MIA PaCa-2 and M-27 control and DFMO-treated cells (2.5 mM and /or 5 mM as specified in the Results section) were collected 48 h and 96 h after subculture by lysing on ice in radioimmunoprecipitation assay (RIPA) buffer (PBS, 1% NP-40, 0.5% sodium deoxycholate, 0.1% SDS, 30 µg/ml aprotinin, 100 mM sodium orthovanidate, and a 10 mg/ml phenylmethylsulfonyl fluoride).

fluoride). Samples were kept on ice for 30 min, followed by centrifugation at 13,000 × g for 10 min. Supernatants were collected and protein concentration was determined using the Bio-Rad DC protein assay. Fifty micrograms of cell lysate was loaded per lane and run on Any-kd™ precast gels (Criterion TGX Stain-free, Cat.#5678123, Bio-Rad Laboratories, Inc., CA) using Bio-Rad Criterion Cell gel apparatus (Cat.#165–6001, Bio-Rad Laboratories, Inc, CA). The proteins were transferred electrophoretically to Hybon-C nitrocellulose membrane (Amersham, Arlington Heights, IL) using Bio-Rad Trans-Blot^®^ SD Semi-dry electrophoretic transfer cell (Bio-Rad Laboratories, Inc., CA). Membranes were blocked in BlottoA (5% w/v nonfat dry milk, 0.1% Tween 20, and Tris-buffered saline (TBS) consisting of 10 mM Tris-HCl, pH 8.0, 150 mM NaCl) for 1 h at room temperature. The primary and secondary antibodies used in this study and blotting conditions are presented in Table S1. The primary antibodies were incubated with the blots overnight at 4 °C. The blots were washed in TBS/0.05% Tween-20. Proteins were detected with enhanced chemiluminescence detection reagent (ThermoFisher Scientific Super Signal^®^ West Pico Chemiluminescent Substrate, Cat#340077). All western blots were repeated at least two times. Image processing program, Image J, version 1.53, was used for evaluation of western blot bands intensity where specified.

### Cell migration and invasion in transwell assay

MIA PaCa-2 control cells and the cells treated with 2.5 mM DFMO were seeded in 24-well Boyden chambers with either control plastic inserts or Matrigel coated inserts with 8.0 μm pore size (BD Biosciences Discovery Labware, Bedford, MA). In the lower chamber of each well 0.5 mL media with FBS was added as a chemoattractant. Cell suspensions were prepared at a concentration of 0.5 × 10^6^ cells/ml in serum-free medium. One hundred microliters of the cell suspensions were plated into inserts. The control and DFMO-treated cells were plated in sextuplet for each condition (migration on control inserts and Matrigel invasion). Cells were permitted to migrate for 48 h. The media was discarded and non-invading cells inside inserts were removed using a swab. Wells were then washed, fixed in 100% methanol for 2 min and stained with 1% Toluidine Blue in 1% borax for 2 min. The stained inserts were washed twice in distilled water and air-dried. The stained inserts were dissolved in 200 µl 0.1 M citric acid in a 96-well plate while shaking for 5 min on a high-speed plate shaker. The supernatant was then transferred to a new well and read at 560 nm on an EL800 Universal Microplate Reader (Bio-Tek Instruments, Inc., Winooski, VT).

### F-actin visualization

MIA PaCa-2 cells were seeded on chamber slides (Lab-Tek; Nalge Nunc International, Naperville, IL) at a concentration of 1 × 10^5^ cells/chamber and were treated with DFMO at the concentration of 2.5mM and 5 mM or vehicle (H_2_O) at the time of subculture. Cells were grown for 96 h after subculture before staining. Immunofluorescence staining for F-actin was carried out using ActinGreen™ 488 ReadyProbes™ reagent according to the manufacturer’s instructions (Cat.#R37110, ThermoFisher Scientific, Inc.). Briefly, cells were washed with phosphate-buffered saline (PBS), fixed in fixative solution (4% formaldehyde in PBS) for 10 min at room temperature, permeabilized with permeabilization buffer (0.5% Triton X-100 in PBS) for 5 minutes at room temperature, washed twice with PBS and stained with ActinGreen 4888 ReadyProbes reagent for 30 min. Cells were photographed with Nikon E800 confocal microscope with epifluorescence and DIC capabilities at 60 x magnification.

### SCID orthotopic mouse model of tumorigenesis

Orthotopic injections and subsequent analyses were conducted on 6–7 weeks old Severe Combined Immunodeficient (SCID) mice (CB-17/IcrACCscid) of both sexes obtained from Taconic Inc. (Germantown, NY). The SCID mice were housed at the animal facility in the Arizona Cancer Center. The facility is AAALAC accredited and holds an Animal Welfare Assurance # D16-00159 (A-3248-01). Mouse colonies were maintained under microisolation and were handled and manipulated only in laminar flow hoods. All procedures involving animals were conducted according to the University of Arizona Animal care and Use Committee policies.

MIA PaCa-2 and M-27 cells at the concentration of 3 × 10^6^ cells in 50 µl Matrigel (Becton Dickenson) were injected into the tail area of the pancreas of anesthetized mice by using a 30-gauge needle. One week after inoculation, animals of each injected cell line were weighed and randomized into two groups (7–9 animals per group). Animals in the control groups were given 0.3 ml of sterilized water daily by gavage. Animals in the treatment groups were given DFMO (500 mg/kg/day by oral gavage). DFMO solution was prepared weekly in sterilized deionized water and stored at 4 °C. All control and treatment groups were fed the irradiated Teklad Global diet 2919 (Envigo RMS Division, Indianapolis, IN, USA) throughout the experiment. Animals were observed twice weekly for adverse signs associated with tumor growth (e.g., > 20% body weight loss, immobility, loss of grooming), at which time mice were euthanized. The overall duration of the experiment was 60 days. The experiment was repeated twice.

### Statistics

Statistical analysis of cell culture experimental data was performed using the paired *t-*test (Microsoft Excel, Microsoft Corp.). The Kaplan-Meier survival curve was estimated by group. The log-rank test was utilized to assess the differences in survival curves. No adjustment for multiple comparisons was performed. The anti-tumor efficacy of DFMO was assessed in animal groups injected with MIA PaCa-2 and M-27 pancreatic cell lines by calculating the percent increase in median survival rate within each cohort (MIA PaCa- 2 and M-27 animals) via the following steps: 1- the median animal survival times in control groups were subtracted from the median survival times in treated groups;2- the difference was divided by the median survival time of animals in control groups; 3-the obtained number was multiplied by 100%.

## Results

### Effects of DFMO treatment on growth and intracellular polyamine content in MIA PaCa-2 and M-27 pancreatic cell lines

**Fig. 1:**
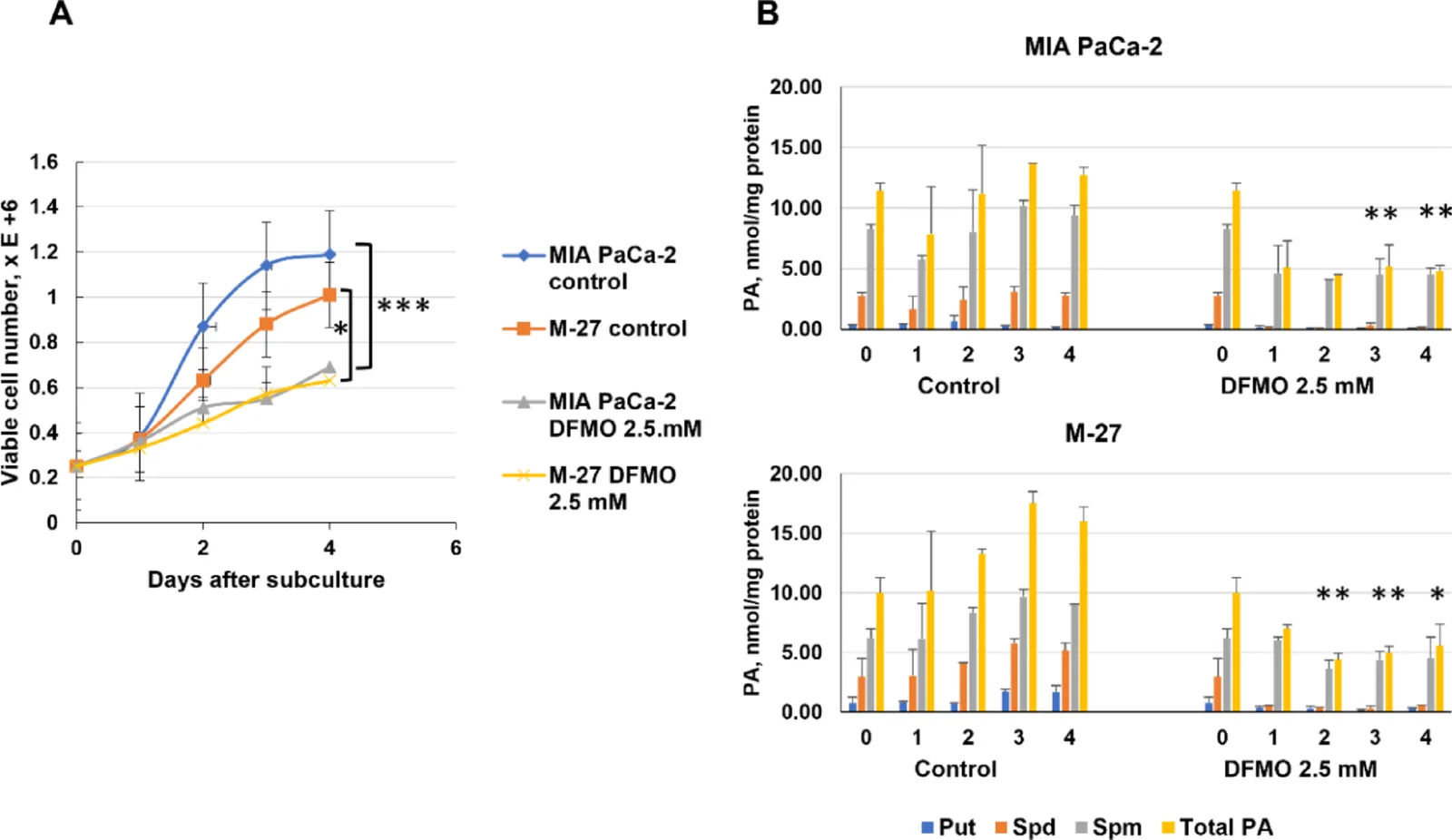
Effects of DFMO treatment on MIA PaCa-2 and M-27 cell growth and polyamine content. Cells were seeded in media containing vehicle (H_2_O), or 2.5mM DFMO and harvested on days 1,2,3, and 4 after subculture for cell counting using a Beckman Coulter Vi-Cell™ Cell Viability Analyzer (**A**) and polyamine analysis by HPLC (**B**). Each point is an average of three independent experiments. The paired t-test was used to analyze statistical significance between the control and treatment groups. *p ≤ 0.03, **p < 0.01, ***p < 0.001

We evaluated the growth of MIA PaCa-2 and M-27 pancreatic cells after subculturing them in the control vehicle (H_2_O) containing media, or in media containing DFMO at a concentration of 2.5 mM for up to 96 h. The growth rates of the control MIA PaCa-2 and M-27 cells were not statistically significantly different, although M-27 cells grew slightly slower than MIA PaCa-2 cells. The DFMO-treated cultures of MIA PaCa-2 and M-27 cells grew significantly slower than control cells (*p* < 0.001 and *p* ≤ 0.03, respectively, by Day 4 after subculture). Notably, the inhibitory effect of DFMO on MIA PaCa-2 cells was statistically significant starting on Day 2 after subculture (*p* < 0.001). In contrast, in M-27 cells, a statistically significant decrease in growth was observed starting at Day 3 after subculture (*p* < 0.03). The growth rates of DFMO-treated MIA PaCa-2 and M-27 cell lines were similar (Fig. 1A). Analysis of total and viable cell numbers at Days 1, 2, 3, and 4 after subculture showed that DFMO treatment did not significantly affect cell viability (data not shown).

The intracellular polyamine levels were similar in control MIA PaCa-2 and M-27 cells, indicating resistance of the polyamine metabolic pathway to inhibition of mutant K-RAS GTPase activity. DFMO suppressed putrescine and spermidine levels in MIA PaCa-2-treated cells on average by more than 10-fold starting on Day 1 after subculture (Fig. 1B), and the total polyamine content was significantly inhibited in DFMO-treated MIA PaCa-2 cells on Days 3 and 4 after subculture (*p* < 0.01). Similarly, DFMO inhibited putrescine and spermidine levels in M-27-treated cells by more than 6-fold on Day 1 after subculture, leading to suppression of the total polyamine content starting at Day 2 after subculture (*p* < 0.01).

### Analysis of K-RAS downstream signaling pathways in DFMO-treated MIA PaCa-2and M-27 cells

**Fig. 2:**
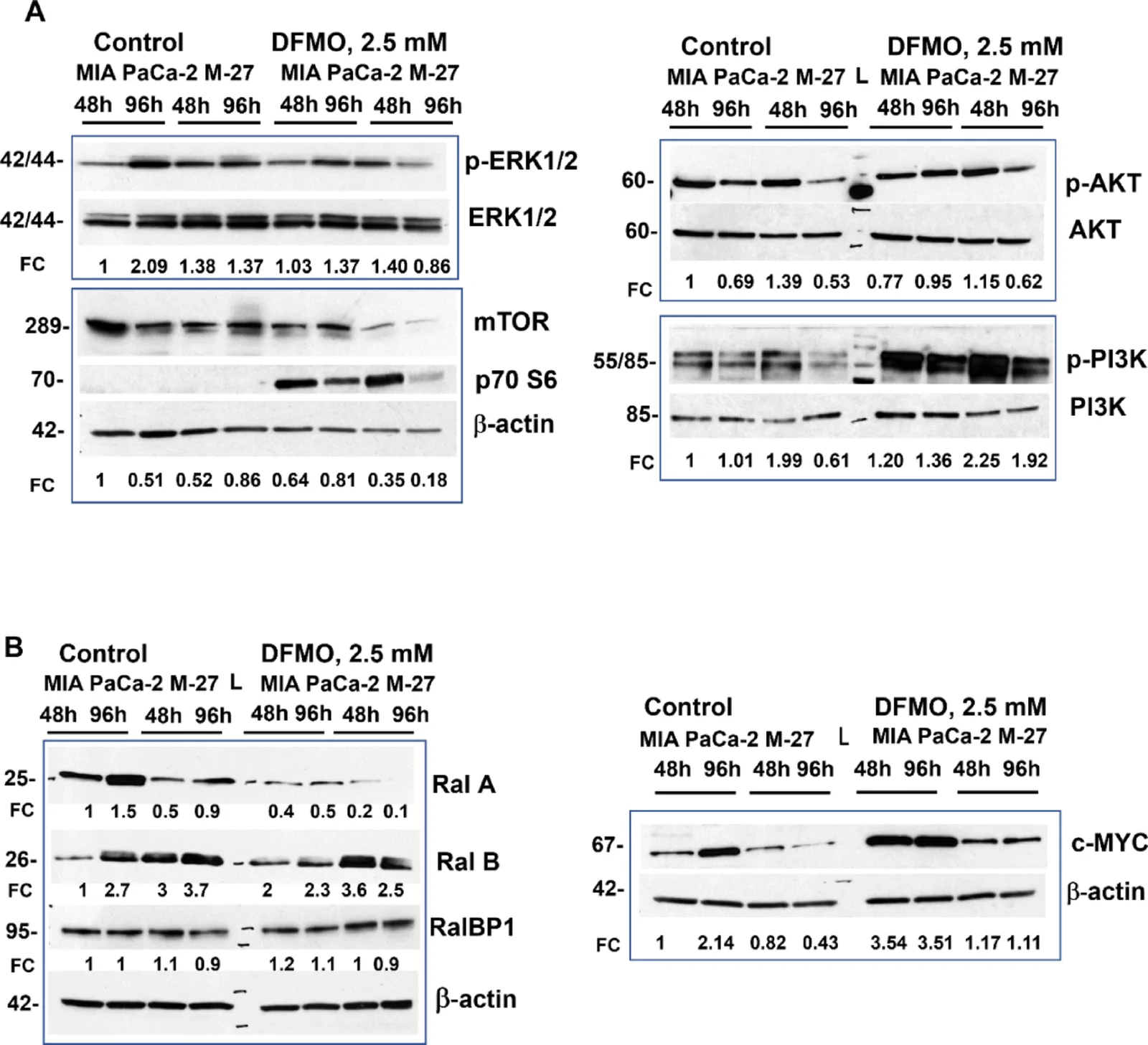
Analysis of signaling pathways downstream of mutant K-RAS in MIA PaCa-2 and M-27 total cell lysates treated with DFMO at a concentration of 2.5 mM by Western blotting. **A** Expression levels of ERK1/2, AKT, PI3K proteins and their phosphorylated forms (p-ERK1/2, p-AKT. p-PI3K), as well as mTOR and p70S proteins in control and treated cells at 48 h and 96 h after subculture. **B** Expression of RalA, and RalB small GTPase proteins, and RalB1 protein (left panel) and c-MYC protein (right panel) in control and DFMO-treated MIA PaCa-2 and M-27 cells at 48 h and 96 h after subculture. L indicates ladder. The bands’ intensities were quantified using ImageJ. Intensities of the bands of the phosphorylated proteins were normalized to the intensities of the bands of total proteins. The intensities of mTOR, RalA, RalB, RalBP1 and c-MYC protein bands were normalized to the intensity of β−actin bands. The fold changes (FC) in bands’ intensities relative to the intensity of the MIA PaCa-2 control band at 48 h is included. The figure is representative of two independent experiments

 As we previously reported, disruption of the K-RAS gene in the M-27 clone through homologous recombination resulted in the loss of RAS activity, which led to a reduction of the downstream K-RAS signaling pathways, a decrease in c-MYC protein level, and suppression of cell migration and invasion (Padavano et al. 2015). Here, we compared the expression of the phosphorylated forms of proteins in the canonical mutant K-RAS signaling pathways: RAC-MAPK-ERK and PI3K-AKT-mTOR (Young et al. 2009) in the control and DFMO-treated MiaPaCa-2 and M-27 cell lines. We measured the protein levels of phosphorylated extracellular signal-activated kinases 1 and 2 (ERK1/2), phosphoinositide 3-phosphate lipid kinase (PI3K), protein kinase B (AKT), and the mammalian target of Rapamycin (mTOR) in cells cultured in the presence of 2.5 mM DFMO for 48 h and 96 h (Fig. 2A). In MIA PaCa-2 cells treated with 2.5 mM DFMO, the most significant inhibitory effect was noted on the ERK1/2 signaling pathway, as evident by an approximately 2-fold suppression in the level of phosphorylated ERK1/2 (p-ERK1/2) protein in cells treated for 96 h. The level of suppression of p-ERK1/2 activation in DFMO-treated MIA PaCa-2 cells was comparable to that of the control M-27 cells (Fig. 2A, *left panel*). DFMO treatment did not significantly alter the level of phosphorylated AKT in MIA PaCa-2 and M-27 cell lines. DFMO treatment slightly increased the level of phosphorylated PI3K (p-PI3K) in MIA PaC-2 cells. In M-27 cells, p-PI3K level was 2-fold higher than in MIA PaCa-2 cells and DFMO treatment maintained this level of induction at both 48 h and 96 h after subculture (Fig. 2A, *right panel*). The level of mTOR protein was not significantly atletered in DFMO-treated MIA PaCa-2 cells but was 2–4 times lower in DFMO-treated M-27 cells at 48 h and 96 h aftersubculture, respectively. The phosphorylated form of S6 protein (p70 S6), which is a downstream target of mTOR kinase, was detected only in DFMO-treated MIA PaCa-2 and M-27 cells (Fig. 2A, *left panel*).

The RAS-like oncoproteins A and B (RalA and RalB) are also known important downstream effectors of mutant K-RAS (Urano et al. 1996). We measured levels of RalA, RalB, as well as their downstream effector, Ral Binding protein (RalBP1), in MIA PaCa-2 and M-27 cells treated with 2.5 mM DFMO. The RalA protein level was suppressed by 2-fold at 48 h and by more than 3-fold at 96 h in DFMO-treated MIA PaCa-2 cells, whereas in M-27 cells RalA protein level was suppressed by more than 2-fold at 48 h and 5-fold at 96 h compared to the corresponding controls. The expression of the RalB protein elevated by 2-fold in DFMO-treated MIAPaCa-2 cells. The level of RalB protein was elevated in control M-27 cells by 3 fold compared to MIA PaCa-2 control cells and DFMO treatment did not alter it further (Fig. 2B). The level of RalBP1, however, was not significantly altered by DFMO (Fig. 2B). Expression of RalA-regulated proteins, EXO84 and SEC5, which are members of the exocyst complex (Moskalenko et al. 2003) and contribute to Ral-mediated endocytosis and RAC activity (Fenwick et al. 2010), was not affected by the DFMO treatment (data not shown).

The c-MYC oncogene is a known downstream target of the RAS-MAPK-ERK pathway and an established transcriptional regulator of the ornithine decarboxylase (*ODC*) gene (He et al. 1998; Fultz and Gerner 2002). Western blot analysis of c-MYC levels in MIA PaCa-2 and M-27 cell lines demonstrates that c-MYC level was inhibited in the control M-27 cell line compared with MIA PaCa-2 cells by 5-fold at 96 h after subculture. DFMO treatment induced c-MYC levels in both cell lines by 3 fold at 96 h after subculture (Fig. 2B, *right panel*). ODC expression was not altered in MIA PaCa-2 and M-27 cells treated with DFMO, which indicates that changes in c-MYC levels do not modify the ODC protein level in DFMO-treated pancreatic cancer cells, while inhibiting the enzyme’s activity (Supp. Figure S1).

### Effect of DFMO treatment on K-RAS protein expression in MIA PaCa-2 cells

**Fig. 3:**
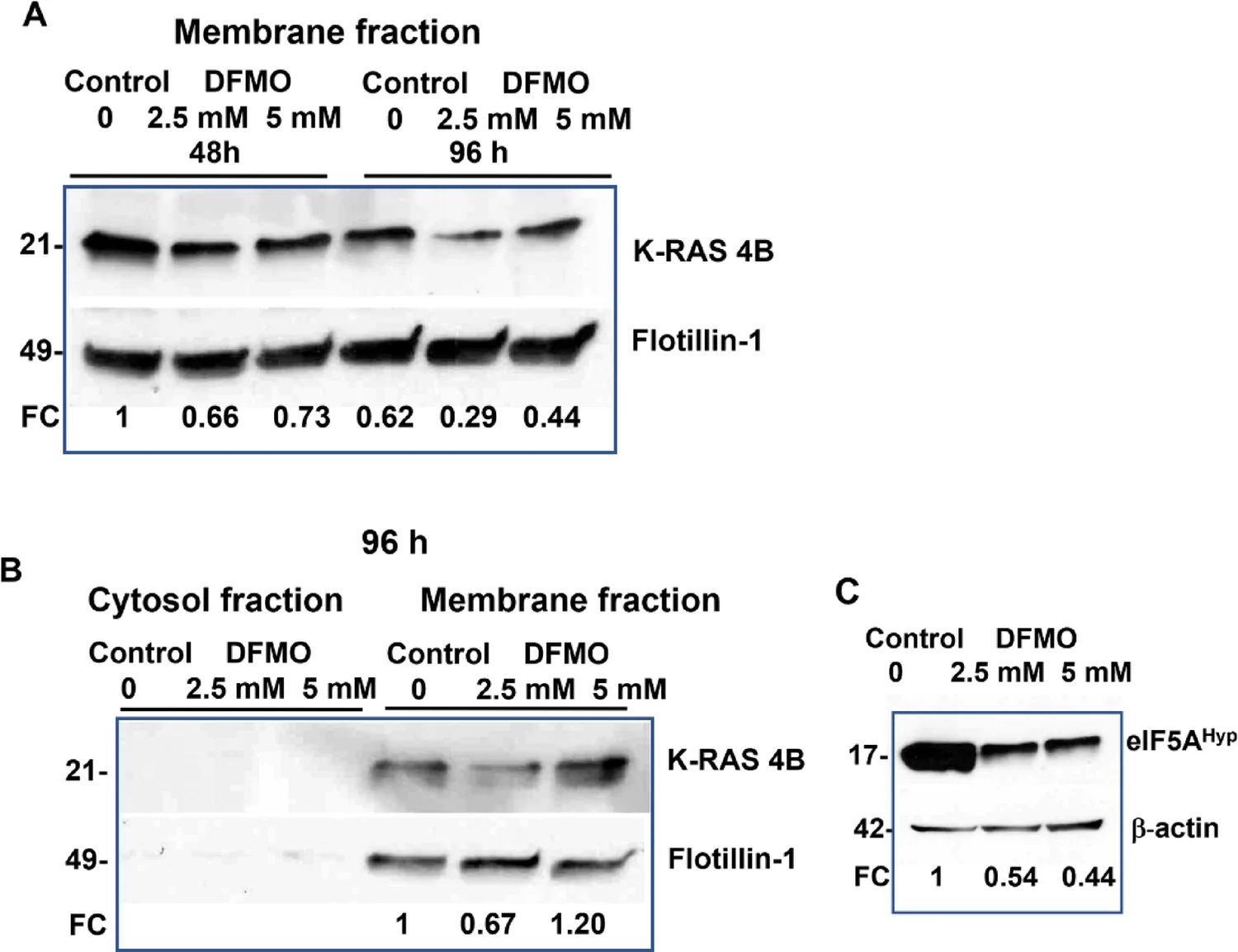
Analysis of K-RAS expression in MIA PaCa-2 cells treated with DFMO at the concentrations of 2.5 mM or 5 mM. by Western blotting. **A** Analysis of K-RAS protein level in the membrane fraction at 48 h and 96 h post-treatment with DFMO at the concentrations of 2.5 mM or 5 mM. **B** K-RAS protein levels in the cytosol and membrane fractions of MIA PaCa-2 cells at 96 h after subculture in control and cells treated with DFMO at the concentrations of 2.5 mM and 5 mM. The intensity of the bands was quantified using ImageJ and normalized to the intensity of flotillin 1 bands. The fold changes (FC) in the intensities of K-RAS bands in the treated cells relative to their levels in the control cells at 48 h (**A**) and 96 h (**B**) are shown. **C** Levels of hypusinated eIF5A (eIF5A^Hyp^) in MIA PaCa-2 cells treated with 2.5 mM or 5 mM DFMO for 48 h. Bands’ intensities were quantified using ImageJ and normalized to the intensity of β−actin bands. The fold changes (FC) in band intensities of eIF5A^Hyp^ in MIA PaCa-2 cells treated with DFMO-are shown. The figures are representative of two independent experiments

 To exert its biological function, the K-RAS protein must be localized to the cell membrane (Zhou et al. 2017). Therefore, we assessed the level of K-RAS protein (K-RAS 4B splice variant) in the membrane fractions of MIA PaCa-2 cells. Cells were cultured in DFMO-containing media at the concentrations of 2.5 mM or 5 mM and were collected for Western blot analysis at 48 h and 96 h after subculture. We observed a time-dependent decrease in K-RAS protein level in the membrane fraction of DFMO-treated MIA PaCa-2 cells compared to its level in control cells, with the most notable decrease in cells treated with 2.5 mM of DFMO at 96 h (2-fold decrease compared to control cells at 96 h and 3.4-fold decrease compared to control cells at 48 h (Fig. 3A). Additionally, we measured the level of K-RAS protein in the cytosol and membrane fractions of MIA PaCa-2 cells treated with 2.5 mM or 5 mM DFMO for 96 h. We detected K-RAS protein mostly in the membrane fraction of control and treated cells, but not in the cytosol fraction. We observed a 2-fold suppression of K-RAS protein level in cells treated with DFMO at the concentration of 2.5 mM, while 5 mM did not change the K-RAS protein level in the membrane fraction (Fig. 3B).

Polyamines play a significant role in cell growth because of their polycationic nature and also because triamine spermidine is a precursor of the rare amino acid hypusine, which is found uniquely in the eukaryotic translation initiation factor 5 A (eIF5A) (Park and Wolff 2018). We measured the level of the hypusinated eIF5A (eIF5A^hyp^) in MIA PaCa-2 cells treated with 2.5 mM or 5 mM DFMO for 48 h and found that both concentrations suppressed the eIF5A^hyp^ level, compared to control cells by 2 fold (Fig. 3C).

### DFMO suppressed F-actin polymerization and the EMT-promoting transcription factor Snail

**Fig. 4:**
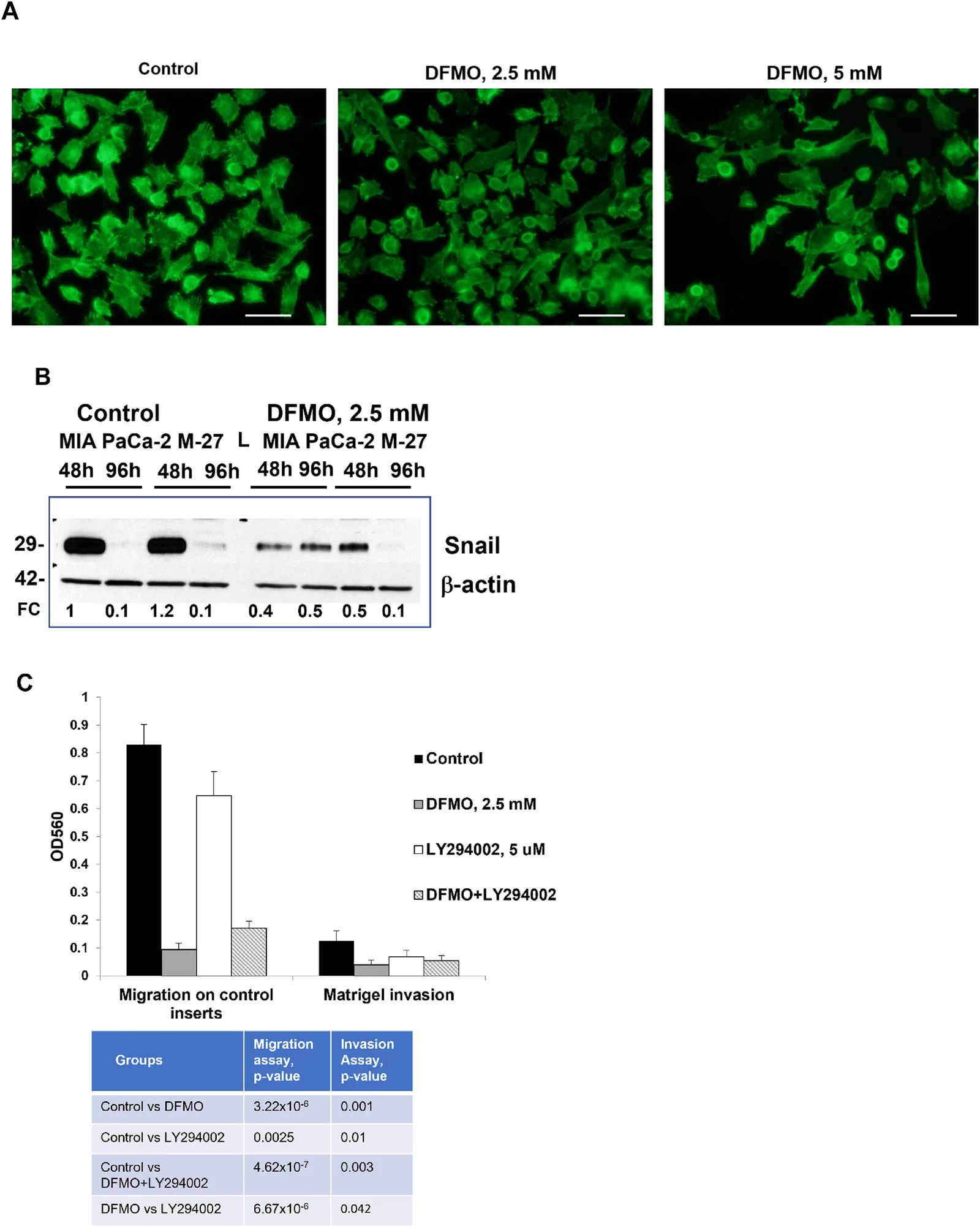
DFMO suppresses filamentous (F)-actin formation and Snail expression in MIA PaCa-2 cells. **A** Representative confocal microscope images of immunofluorescence staining for F-actin at 96 h post-treatment with DFMO at the concentrations of 2.5 mM and 5 mM. The scale bar is 500 µM. **B** Western blot analysis of Snail in control and DFMO-treated cells at 48 h and 96 h after subculture. L indicates ladder. The bands’ intensities were quantified using ImageJ and normalized to the intensity of β−actin bands. The fold changes (FC) in the intensity of each band relative to the intensity of the MIA PaCa-2 control band at 48 h are included. **C** Evaluation of DFMO (2.5mM) and LY294002 (50 µM) treatments on MIA PaCa-2 cell migration and invasion at 48 h after subculture. The paired *t*-test was used to analyze statistical significance between treatment groups and p-values of the differences between the control and different treatments are presented in the table. In each experiment, control and treated cells were plated in sextuplets for each condition. The figure is representative of two independent experiments

 As we reported above, DFMO treatment suppressed the level of RalA small GTPase in MIA PaCa-2 cells, while having a limited effect on RalB GTPase. It is known that RalA GTPase, but not RalB GTPase, binds filamin, resulting in the formation of filopodia via direct polymerization of F-actin, which increases membrane protrusions and promotes cell migration (Yang et al. 2016; Ohta et al. 1999). We visualized F-actin in control and DFMO-treated MIA PaCa-2 cells at 96 h after subculture using AlexaFluor™488 phalloidin probe, which selectively labels F-actin. F-actin was visible in cell protrusions in control cells but absent in cells treated with either 2.5 mM or 5 mM DFMO (Fig. 4A).

Cell migration also depends on the formation of new focal contacts and the reorganization of the cytoskeleton (Yamada and Miyamoto 1995). We measured the level of expression of phosphorylated focal adhesion kinase p125^FAK^ (FAK), which indicates activation of focal contacts. The levels of phosphorylated FAK protein in lysates of the MIA PaCa-2 cells treated with 2.5 mM DFMO were similar to those of the control cells (data not shown). These data argue that DFMO does not influence FAK phosphorylation in the MIA PaCa-2 pancreatic cancer cell line.

Next, we measured the level of Snail protein in the control and DFMO-treated MIA PaCa-2 and M-27 cell lines. Snail is a major transcription factor upregulated in the process of the epithelial-mesenchymal transition (EMT) during pancreatic intraepithelial neoplasia (PanIN) progression (Fendrich et al. 2018). The control MIA PaCa-2 and M-27 cells expressed comparably high levels of Snail protein at 48 h after subculture, following a 5-fold suppression at 96 h after subculture. DFMO-treated MIA PaCa-2 cells expressed 2 times less Snail protein at both 48 h and 96 h. The Snail protein level in DFMO-treated M-27 cells was 6-fold lower at 48 h after subculture compared to the untreated cells (Fig. 4B).

Next, we measured the migration and invasion of MIA PaCa-2 cells in vitro. Cells were seeded in Boyden chambers on uncoated plastic inserts for the cell migration assay and in Matrigel-coated inserts for the assessment of cell invasion. After seeding, the cells were treated with DFMO at 2.5 mM or with potent PI3K inhibitor LY294002 at 50 µM for 48 h alone or in combination. We found that DFMO, as a single agent, suppresses cell migration and invasion more effectively than LY294002 (Fig. 4C, DFMO vs. LY294002 *p* = 6.67 × 10^− 5^, cell migration assay, *p* = 0.042, cell invasion assay).

### DFMO suppresses pancreatic tumorigenesis and prolongs animals’ survival in vivo

**Fig. 5:**
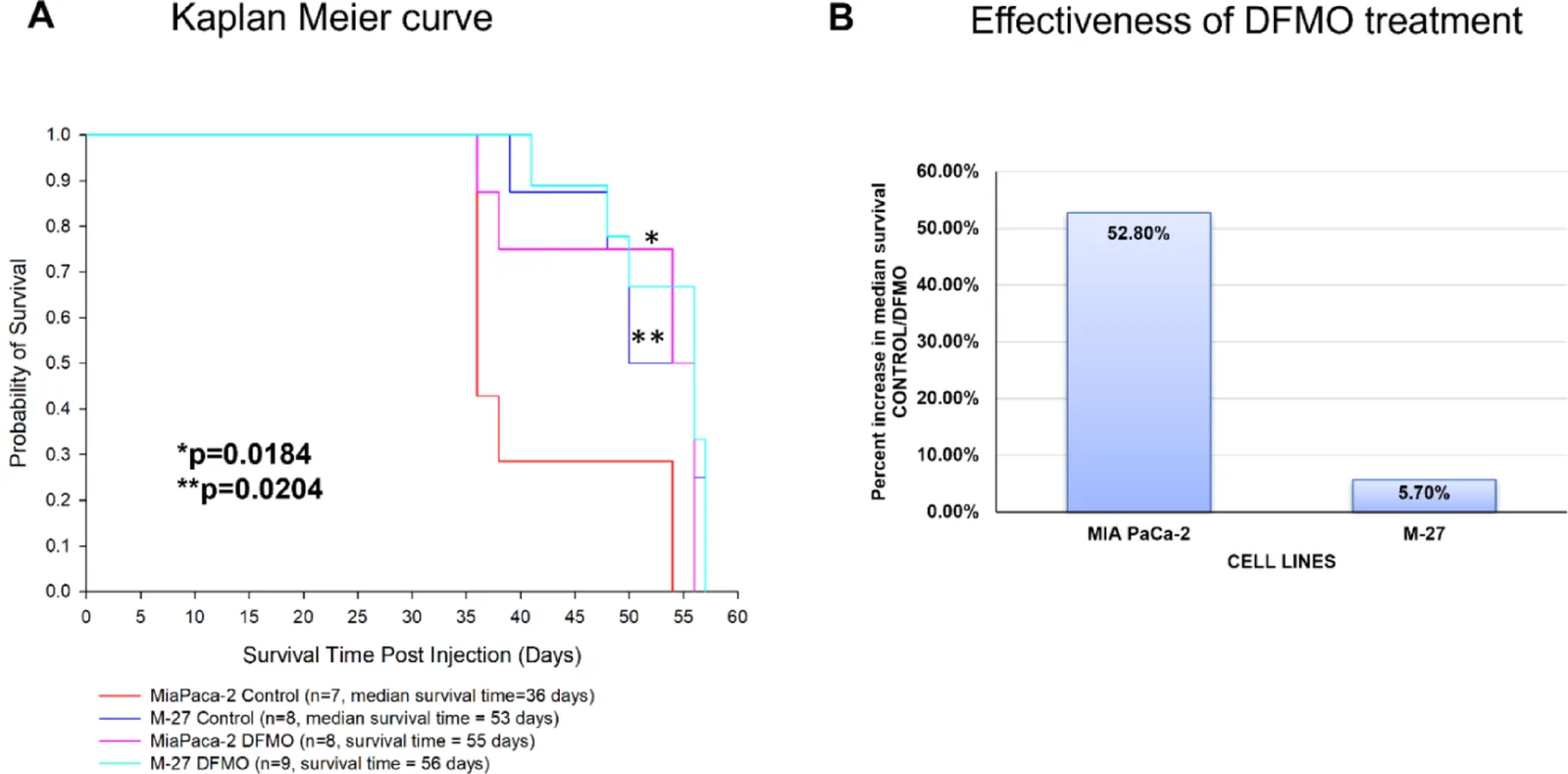
Analysis of anti-tumor DFMO activity in vivo using a mouse orthotopic pancreatic cancer model. **A**. Kaplan- Meier survival curves of animals injected with MIA PaCa-2 and M-27 cells, which received the vehicle (Control groups) or DFMO (500 mg/kg/day by oral gavage). Treatment started one week post-implantation of tumor cells into the pancreas. Statistical significance between the control and treated groups was determined using the log-rank test. **B.** Analysis of DFMO treatment effectiveness in animals with MIA PaCa-2 and M-27 tumors

 Finally, we assessed the antitumor effect of DFMO treatment using the orthotopic pancreatic cancer mouse model. MIA PaCa-2 and M-27 cells were injected into the pancreas of SCID mice at a concentration of 3 × 10^6^ cells. An equal number of male and female mice per group was used. One week after cell inoculation, animals were assigned to the control (MIA PaCa-2, *n* = 7; M-27, *n* = 8) and treatment (MIA PaCa-2 DFMO, *n* = 8; M-27 DFMO, *n* = 9) groups. Animals in the treatment groups received DFMO at a dose of 500 mg/kg/day by oral gavage, while the control animals received sterilized deionized water (vehicle) by oral gavage. The DFMO dose in this experiment was equivalent to the human dose of 1500 mg/kg/day (Nair and Jacob 2016). There was no significant change in animals’ weight between control and treatment groups throughout the duration of the experiment. However, the average weight of animals injected with M-27 cells was statistically significantly lower than that of animals injected with MIA PaCa-2 at the time of sacrifice (Figure S2, *p* < 0.05). The median survival time of DFMO-treated MIA PaCa-2 tumor-bearing animals was, on average, 1.5 times longer than that of the control group (median survival time 53 days for DFMO-treated versus 36 days for vehicle-treated groups, MIA PaCa-2 control vs. MIA PaCa-2 DFMO, *p* = 0.0184). Similarly, the M-27 tumor-bearing animals had a statistically significant increase in survival compared to MIA PaCa-2 tumor-bearing mice (MIA PaCa-2 control vs. M27 control, *p* = 0.0204) (Fig. 5A; Table 1). DFMO treatment did not significantly improve the survival rates of mice injected with the M-27 cell line (median survival time of 56 days for DFMO-treated versus 53 days for control animals, *p* = 0.6055). Also, no significant difference in survival rates was found between the DFMO-treated MIA PaCa-2 and DFMO-treated M-27 groups (MIA PaCa-2 DFMO vs. M-27 DFMO, *p* = 0.184). Figure 5B demonstrates that MIA PaCa-2-injected animals responded better to DFMO treatment than M-27-injected animals (52.80% increase in median survival times for MIA PaCa-2-treated animals compared to 5.70% increase for M-27-treated animals).

**Table 1:** The Statistical Significance of the Difference in Survival between Groups

	Median Survival (days)	*p*-value for the difference			
		MIA PaCa-2 control vs. Mia PaCa-2 DFMO	MIA PaCa-2 control vs. M-27 control	M-27 control vs. M-27 DFMO	MIA PaCa-2 DFMO vs. M-27 DFMO
MIA PaCa-2 control	**36**				
M-27 control	**53**		0.0204		
MIA PaCa-2 DFMO	**53**	0.0184			
M-27 DFMO	**56**			0.6055	0.1841

## Discussion

Multiple genetic abnormalities have been identified in pancreatic cancer, with the majority of pancreatic adenocarcinomas (more than 90%) harboring gain-of-function mutations in the *K-RAS* oncogene (Lu and Zeng 2017). Mutant K-RAS plays a major role in neoplastic transformation and cancer progression in the pancreas. Therefore, improving the treatment of PDAC with therapeutically relevant targeted agents is of utmost importance. Efforts to develop targeted therapies have led to the design of novel, effective inhibitors of mutant K-RAS, broadening treatment options for PDAC patients (Huang et al. 2021; Holderfield et al. 2024).

Dependence of pancreatic cancer cells on polyamines for growth and invasion led to the development of a polyamine-targeting strategy using the FDA-approved inhibitor of polyamine biosynthesis DFMO and other polyamine pathway modulating agents. The results of experimental studies suggest that this is a promising approach to delay or inhibit pancreatic cancer progression (Mohammed et al. 2014; Gitto et al. 2018).

Here, we tested the molecular effects of DFMO in MIA PaCa-2 cells and its isogenic cell line, M-27, which lacks the constitutive activation of RAS protein. Accumulation of the GTP-bound RAS (also known as RAS (ON), which drives aberrant signaling, was eliminated in the M-27 cell line through homologous recombination, and the molecular and phenotypic consequences of this genetic alteration were previously characterized (Padavano et al. 2015). In the current study, we initially analyzed the growth rates and polyamine content of MIA PaCa-2 and M-27 cell lines. We learned that disruption of mutant K-RAS activity in M-27 pancreatic cancer cells did not significantly alter their growth rates and the intracellular polyamine levels compared with the MIA PaCa-2 cell line. This observation indicates that M-27 cells maintain dependency on RAS signaling possibly through other genomic amplification or alterations in upstream or downstream signaling pathways (Singh et al. 2023). On the contrary, DFMO treatment effectively suppressed the growth and polyamine levels in both MIA PaCa-2 and M-27 cell lines.

Analysis of the expression patterns of proteins that respond to the mutant K-RAS signaling showed that DFMO suppressed phosphorylation of ERK1/2. At the same time, the PI3K signaling was induced in DFMO-treated MIA PaCa-2 and M-27 cell lines, which may be a compensatory mechanism in response to the lack of oncogenic stimuli. In the MIA PaCa-2 cells, DFMO was more effective in suppressing pancreatic cell migration and invasion in vitro than the PI3K inhibitor LY294002. This observation corroborates the previously reported resistance of the MIA PaCa-2 cell line to treatments with gemcitabine (He et al. 2024) and LY294002, which also increased PI3K phosphorylation (Wang et al. 2017).

The mTOR protein level was inhibited in DFMO-treated M-27 cells while no significant change in its expressionlevel was seen in DFMO-treated MIA PaCa-2 cells, with concomitant induction of the phosphorylated form of ribosomal p70 S6 kinase. This kinase is a downstream target of mTOR, and its phosphorylation indicates possible mTOR resistance to DFMO treatment and reactivation of p70 S6 kinase by alternative kinases, such as PI3K.

We further found that DFMO treatment significantly induced c-MYC protein levels in both MIA PaCa-2 and M-27 cells, although it did not increase the expression of its target protein ODC. C-MYC contributes to tumorigenesis through multiple mechanisms. Particularly, it is a known regulator of several transcription factors, which promote EMT (Liu et al. 2013). In our cell models, DFMO treatment inhibited expression of the EMT transcriptional factor Snail and cell migration-inducing small GTPase RalA protein. DFMO also suppressed F-actin polymerization in treated MiaPaCa-2 cells.

The combined changes in expression of oncogenic RAS signaling proteins resulting from DFMO treatment in MIA PaCa-2 and M-27 cell lines are summarized in Table 2. Collectively, they induced suppression of the migratory phenotype of MIA PaCa-2 cells, which we confirmed using *in vitro* migration and invasion assays.

**Table 2:** Summary of DFMO-dependent changes in expression of mutant K-RAS signaling proteins in MIA PaCa-2 and M-27 cells

Signaling molecules	MIA PaCa-2 + DFMO	M-27 + DFMO
p-ERK1/2	Reduced ++	Reduced +
p-PI3K	Induced +	Induced++
p-AKT	no effect	no effect
mTOR	no effect	Reduced+++
Ral A	Reduced+++	Reduced++++
RalBP1	no effect	no effect
c-MYC	Induced+++	Induced+++
Snail	Reduced++	Reduced++

+ indicates fold change at 96 h after subculture

Furthermore, we observed that DFMO treatment suppressed the level of hypusinated eIF5A in MIA PaCa-2 cells. The eIF5A translation factor regulates the expression of many proteins, including those involved in cytoskeleton formation, cell migration, and invasion in pancreatic cancer cells (Fujimura et al. 2015). Hypusination of eIF5A is necessary for the function of this translation factor and requires the synthesis of the unique amino acid hypusine (N-(4-amino-2-hydroxynutyl)lysine) via addition of the butylamine portion of spermidine (Park 2006). We observed a 2-fold suppression in the level of eIF5A^Hyp^ in DFMO-treated MIA PaCa-2 cells, presumably because of the significant decrease in the intracellular spermidine levels. Suppression of eIF5A hypusination in the PDAC cell line PANC-1 using the spermidine analog GC7 inhibited cell migration and invasion in cell culture and in orthotopic mouse models (Fujimura et al. 2014). Therefore, the decrease in eIF5A hypusination in MIA PaCa-2 cells upon DFMO treatment contributes to the mechanisms of DFMO anti-tumor activity.

Additionally, we measured the total K-RAS protein level in control and DFMO-treated MIA PaCa-2 cells. The wild-type K-RAS protein is functionally active at the plasma membrane, where it switches from its active GTP-bound state to an inactive GDP-bound state due to GTP hydrolysis (Trahey and McCormick 1987). We found that the level of total K-RAS protein in the membrane fraction of DFMO-treated cells decreased compared to its level in the membrane of control cells. It has been previously reported that expression of K-RAS oncogene is negatively regulated by the let-7 family of miRNAs (Johnson et al. 2005). Polyamine depletion in colon cancer cells altered multiple mRNAs, and, particularly, caused the upregulation of the let-7 family of mRNAs (Paz et al. 2014). The observed decrease in K-RAS protein level could be mediated by let-7i mRNAs in DFMO-treated MIA PaCa-2 cells. Since we did not find changes in the levels of exocyst marker proteins in DFMO-treated cells, we cautiously suggest that DFMO did not alter the trafficking of K-RAS protein to the plasma membrane.

We extended the analysis of the anti-tumor activity of DFMO in pancreatic cancer cells in vitro by testing DFMO efficacy *in vivo* in the SCID mouse orthotopic pancreatic cancer model. Treatment of animals with 500mg/kg/day dose of DFMO, which is equivalent to the dose tested in human clinical trials, significantly improved their survival time, compared to the control group (MIA PaCa-2 control group vs MIA PaCa-2 DFMO group, *p* = 0.0184). The median survival time of animals in the MIA PaCa-2 DFMO group was equal to the median survival time of animals in the M-27 control group (53 days for both groups). This finding demonstrates the efficacy of DFMO against the oncogenic RAS activity in pancreatic cancer cells. We also noted that despite the similar inhibitory effects of DFMO on mutant K-RAS signaling in MIA PaCa-2 and M-27 cell lines (Table 2), DFMO treatment increased the animals’ median survival time in the MIA PaCa-2 DFMO group more significantly (by 52.80%), compared to the M-27 DFMO group (by 5.70%). The significantly smaller increase in survival time of animals in M-27 DFMO group indicates that treatment with the inhibitor of polyamine biosynthesis did not provide additional benefits when the oncogenic activity of RAS protein was eliminated. The M-27 cells may have adapted to the loss of RAS activity through changes in metabolic pathways, which are less sensitive to DFMO.

DFMO could modulate the metabolic pathways in pancreatic cancer cells, which were not evaluated here. Particularly, the immunodeficient orthotopic pancreatic cancer model utilized in this study did not allow us to assess the effects of DFMO on immune functions in tumor-bearing animals. But previous investigation of the effects of DFMO treatment in immunocompetent mouse pancreatic cancer model found that DFMO can reverse the immunosuppressive PDAC tumor microenvironment through the increased infiltration of CD4 + and CD8 + T-lymphocytes (Nakkina et al. 2021).

Overall, the multitargeting antitumor activity of DFMO in pancreatic cancer cells may be used as a therapy when PDAC tumors become resistant to mutant K-RAS inhibitors. SBP-101 (diethyl dihydroxyhomospermine), a polyamine analog that also disrupts polyamine metabolism, is currently under clinical investigation in combination with nab-paclitaxel and gemcitabine in patients with PDAC who have not received prior therapies for PDAC (ASPIRE Phase 2/Phase 3 Clinical Trial Number NCT05254171, Registration date posted 2022-02-24, Clinicaltrials.gov). The findings presented here, and new clinical developments, add to the rationale for using disruptors of polyamine metabolism, alone or in combination with other drugs, to inhibit theprogression of PDAC tumors that express the activated K-RAS oncogene.

## Supplementary Information

Below is the link to the electronic supplementary material.

Supplementary Material 1

## Data Availability

No datasets were generated or analysed during the current study.

## Abbreviations

PDAC: Pancreatic adenocarcinoma
DFMO: α-difluoromethylornithine
SCID mice: Severe combined immunodeficient mice
