# Supplementary material for: Difluoromethylornithine modulates mutant K-RAS signaling pathways in pancreatic cancer cells
Source: Amino Acids. 2026 Sep 16;58:60. doi: 10.1007/s00726-026-03559-5 (PMC13676551; doi:10.1007/s00726-026-03559-5)
Supplement: Supplementary file 1 [file 726_2026_3559_MOESM1_ESM.docx]

**Supplementary Materials**

**Table S1.** List of antibodies.

| **Antibody** | **1^o^ antibody dilution** | **Source** |
| --- | --- | --- |
| K-RAS 4B | 1:200 in Blotto A | sc-30 |
| c-MYC | 1:400 in Blotto A | sc-40 |
| Hypusinated eIF5A | 1:1000 in BlottoA | Millipore ABS1064-I |
| Phospho ERK1/2 | 1:2000 in 5% BSA | CS#4348 |
| ERK1/2 | 1:1000 in 5% BSA | CS#9102 |
| Phospho AKT | 1:500 in 5% BSA | CS#4058 |
| AKT | 1:1000 in 5% BSA | CS#9272 |
| PI3K p85 | 1:1000 in 5 % BSA | CS#4292 |
| Phospho-PI3K | 1:1000 in 5 % BSA | CS#4228 |
| mTOR | 1:1000 in 5% BSA | CS#3570 |
| p70S6 | 1:500 in 5% BSA | CS#9202 |
| SNAIL1 | 1:1000 in 5% BSA | CS#3879 |
| RalA | 1:1000 in 5% BSA | CS#3526 |
| RalB | 1:1000 in Blotto A | CS#3523 |
| RalBP1 | 1:1000 in Blotto A | CS#5739 |
| β-Actin | 1:5000 in 1.5% Blotto A | Sigma A5441 |
| Flotillin-1 | 1:1000 in 5% BSA | CS#3253 |

BlottoA –5% non-fat dry milk in TBST

M- anti-mouse secondary antibody

R-anti-rabbit secondary antibody

CS-Cell Signaling Technology; sc-Santa Cruz Biotechnology Inc.

**Figure S1.** Intracellular level of ODC protein in the control and DFMO-treated MIA PaCa-2 and M-27 cells at 48 h and 96 h after subculture. The Figure is representative of two independent experiments.

**Figure S2**. DFMO treatment does not alter the weights of tumor-bearing mice in the orthotopic pancreatic cancer model. *p<0.05 between the average weights of MIA PaCa-2-injected and M-27-injected animals.
